# Impaired Cerebral Autoregulation in Pediatric Patients with ECMO: A Retrospective Analysis of ECMO Time Periods and Association with Neurologic Injury

**DOI:** 10.1007/s12028-026-02531-1

**Published:** 2026-05-18

**Authors:** Ethan L. Sanford, David R. Busch, Neel Shah, Chasity Custer, Nicolas Joram, Michael Morris, Sumit Singh, Lakshmi Raman

**Affiliations:** 1 Department of Pediatrics, University of Texas Southwestern Medical Center, Dallas, TX USA; 2 Department of Anesthesiology and Pain Management, University of Texas Southwestern Medical Center, Dallas, TX USA; 3 Outcomes Research Consortium, Cleveland, OH USA; 4 Department of Pediatrics, Washington University School of Medicine, St. Louis, MO USA; 5 Department of Pediatric Intensive Care, Nantes University Hospital, Nantes, France; 6 Clinical Investigation, Children’s and Adolescents’ Hospital, Nantes University Hospital, Women’s, Nantes, France; 7 Department of Radiology, University of Texas Southwestern Medical Center, Dallas, TX USA

**Keywords:** Cerebral blood flow, Cerebrovascular circulation, Cerebral autoregulation, Extracorporeal membrane oxygenation, Intensive care units, Pediatric

## Abstract

**Objective:**

Neurologic injury is common in pediatric patients with extracorporeal membrane oxygenation (ECMO). Impaired cerebral autoregulation (CAR) may increase the risk of neurologic injury. We aimed to measure CAR in different phases of ECMO and analyze for association with radiographic neurologic injury.

**Methods:**

We performed a retrospective, single-center, observational cohort study of pediatric (0–18 years) ECMO patients with no prior neurologic injury, cardiopulmonary bypass, ECMO, or congenital heart disease who underwent brain imaging. Correlation of time-matched cerebral oximetry and mean arterial blood pressure (MAP) data determined the cerebral oximetry index (COx) a noninvasive estimate of CAR. Impaired CAR was tested for association with neurologic injury score (NIS) as measured by blinded neuroradiologists. Secondarily, we calculated the area under the curve (AUC) of MAP above or below age-based 50th percentile MAP in temporal windows with impaired CAR. Univariate analysis and a multivariable linear regression model adjusting for type of ECMO, ECMO indication, and age determined the association between impaired CAR and the AUC metric with NIS.

**Results:**

Data from 72 children were analyzed. The median age was 11.7 months, 47 children (65.3%) received VA ECMO, and severe neurologic injury (SNI) occurred in 9 (12.5%). There was no association between percent time with impaired CAR and SNI (16.6% versus 12.2%, *P* = 0.38). Impaired CAR burden was significantly higher pre-ECMO as compared with peri-cannulation and intra-ECMO time periods (26.5% versus 14.8% and 16.6%, *P* < 0.01). The multivariable linear regression model discerned a significant association between pre-ECMO hypotension during impaired CAR and NIS (coefficient = –0.008; –0.015, –0.001; *P* = 0.026; *R*^2^ = 0.19).

**Conclusions:**

Total time with impaired CAR measured by the cerebral oximetry index was not associated with neurologic injury in pediatric patients with ECMO. However, impaired CAR was more common pre-ECMO, and pre-ECMO AUC incorporating magnitude of blood pressure derangement was significantly associated with neurologic injury, implicating pre-ECMO clinical derangements as imparting risk.

**Supplementary Information:**

The online version contains supplementary material available at https://doi.org/10.1007/s12028-026-02531-1.

## Introduction

Extracorporeal membrane oxygenation (ECMO) use for children has increased in the past 12 years with continued increases in use anticipated. The most harmful complications of ECMO are hemorrhagic and ischemic neurologic injuries, which significantly increase the risk of death. Significant injuries occur in 10–25% of pediatric patients with ECMO [1–5]. This likely underestimates the burden of neurologic injury, as up to 70% of pediatric patients with ECMO have at least minor injuries on peri- or post-ECMO head imaging [6–8], and 50% of pediatric ECMO survivors have a new neurologic deficit [9]. Despite increasing use of ECMO, the incidence of related neurologic injuries has not declined. [10–12]

Impaired cerebral autoregulation (CAR) has been implicated as a risk factor for neurologic injury in pediatric patients with ECMO. Normally, cerebral blood flow is constant over a range of blood pressure due to CAR. Cerebral pressure autoregulation is mediated by adaptations of brain blood vessel resistance in response to changes in blood pressure [13]. The disease states of pediatric patients with ECMO cause abnormal blood pressure, metabolic derangements, cerebrovascular inflammation, and the need for sedative medications [2, 14]. These factors may impair CAR. Additionally, ECMO may interrupt CAR due to modulation of blood pressure, rapid changes in acid–base status and oxygenation, and mechanical stress on large blood vessels [15–18]. In pediatric ECMO, the blood pressure at which CAR is impaired likely varies with differences in disease state and patient factors, such as age [19]. The complex interactions of patient, disease, and ECMO factors may prevent application of static blood pressure goals to guarantee appropriate cerebral perfusion pressure in all circumstances. Low blood pressure alone may not predict organ injury. Conversely, a “normal” blood pressure may not be adequate in certain scenarios. The combination of impaired CAR and abnormal blood pressure may compound risk beyond either factor in isolation.

While several methods have been proposed to monitor CAR, none are standard in pediatric critical care, likely because minimal clinical outcomes data exist to support their use. Correlation between cerebral oximetry and blood pressure is termed the cerebral oximetry index (COx). Prior animal studies have validated COx as a surrogate for cerebral autoregulation [20]. Studies in adult patients undergoing cardiac surgery demonstrate worsened outcomes associated with COx-determined impaired CAR [21]. A pilot study of COx index in pediatric patients with ECMO demonstrated an association of impaired CAR with neurologic injury [18]. However, these data are limited to a low number of children with cardiac pathology, in only the intra-ECMO phase of ECMO care. We, therefore, aimed to measure the COx index over the entire episode of care for pediatric patients with ECMO. We hypothesized that the burden of impaired cerebral autoregulation would differ during the pre-, peri-, and intra-ECMO cannulation phases of care and that increased impaired autoregulation is associated with neurologic injury.

## Methods

### Population

We performed a retrospective, observational cohort study of children 18 years old or less cannulated onto venoarterial (VA) or venovenous (VV) ECMO between 1 December 2019 and 1 June 2023 with institutional review board (IRB) approval (STU-2021–0995). Only children who underwent intra-ECMO head computed tomography (CT) or post-ECMO brain magnetic resonance imaging (MRI) and were on ECMO for at least 48 h were included. Children with prior known radiographic neurologic injury, cardiopulmonary bypass within 30 days, prior ECMO, encounters without sufficient data to calculate COx index, and cyanotic congenital heart disease were excluded.

### Neurologic Injury Definition

Patient demographic data and all clinical data were extracted from the electronic medical record and stored in a secure Research Electronic Data Capture (REDCap) database. Intra-ECMO head CT or post-ECMO brain MRI were reviewed by two blinded neuroradiologists who performed image-based scoring of injuries, termed the neurologic injury score (NIS). The NIS is a weighted scoring of parenchymal lesions, hemorrhage, and ventricular dilation. NIS ranges from 0 to 30, with 0 meaning no abnormalities and 30 being the worst possible neurologic injury. For patients with multiple images, MRI was scored if the image was performed within 2 weeks of ECMO decannulation; otherwise, the head CT most proximal to ECMO decannulation was used. The scoring system was initially developed to assess neurologic injuries among neonatal patients on ECMO using ultrasound, CT [22], and MRI [23]. The category descriptions have been modified to add commonly seen injury types in ECMO (e.g., subdural and subarachnoid hemorrhage) [24]. Retrospective analysis showed this system can discriminate severe injuries (NIS > 9) as being associated with significantly worse Pediatric Cerebral Performance Category (PCPC) scales in patients with both venoarterial (VA) and venovenous (VV) ECMO [24]. Severe neurologic injury (SNI) is defined, therefore, as NIS > 9. Any neurologic injury is defined as NIS > 0.

### Cerebral Oximetry Index Calculation

All patients were monitored per standard of care in the intensive care unit (ICU). Cerebral oximetry is standard during ECMO care at the study center, but variable in application before ECMO cannulation. Patients with arterial line and cerebral oximetry data during ECMO were identified. The near-infrared spectroscopy device used was the Medtronic INVOS 5100C regional oximeter routed to the Phillips MX850 patient monitor that is connected to the T3 etiometry platform. The INVOS infant-neonatal sensor (< 10 kg), pediatric sensor (10–40 kg), and 5100C adult sensors (> 40 kg) were used. The NIRS device uses the manufacturer’s proprietary algorithms for signal smoothing, which may attenuate or shift signal oscillations and affect COx estimation. Paired blood pressure and cerebral oximetry data were extracted with the T3 etiometry platform with a time resolution of 5 s. Blood pressure data were filtered to a physiological range (20 ≤ MAP ≤ 120 mmHg), and rate of change was used in both blood pressure and cerebral oximetry to eliminate erroneous rapid changes, e.g., from blood draws, repositioning NIRS [25]. In each 5-min window with at least 20 data points, a Pearson’s correlation coefficient was calculated between the blood pressure and cerebral oximeter output to render COx. As per prior studies [20], consecutive, paired, 10-s averaged values (from 5-s raw data) of 300-s duration were used to calculate each individual COx value. These calculations were made in a moving 60-s window. That is, every 60 s a new value was calculated by integrating 60 s of new data and dropping the trailing 60 s. The COx cutoff for impaired CAR has not been established and may differ by age and pathology. Among infants, higher thresholds (0.4) have been used [26], though several studies in pediatrics and adults have used a threshold of 0.3. A nascent study validating COx against laser-Doppler-derived autoregulation in a piglet model found greatest sensitivity and specificity at a COx threshold of 0.36 [19]. Similarly, COx thresholds were tested for association with impaired autoregulation in adult patients undergoing cardiac surgery who had standard Lassen curves developed from transcutaneous Doppler measurements. A threshold of 0.35 was found to be optimal [27]. The threshold of 0.35 was used in studies documenting a significant association of impaired autoregulation with morbidity and mortality in patients undergoing cardiac surgery [21]. These data were considered in choosing the threshold of 0.35. Data were then stratified into pre- and peri-cannulation, and intra-ECMO epochs defined as 0–24 h prior to ECMO, the first 12 h of ECMO, and 12 h to decannulation or 14 days of ECMO. Data missingness may be due to lapses in monitor application, errors or delays in attaching monitors appropriately, or disconnections of monitoring devices. Time without monitoring was dropped from analysis rather than imputed, as physiologic state during those time periods may vary.

### Area under the Curve of Patient MAP Versus Age-Based 50th Percentile MAP with and without Impaired CAR

**Fig. 1:**
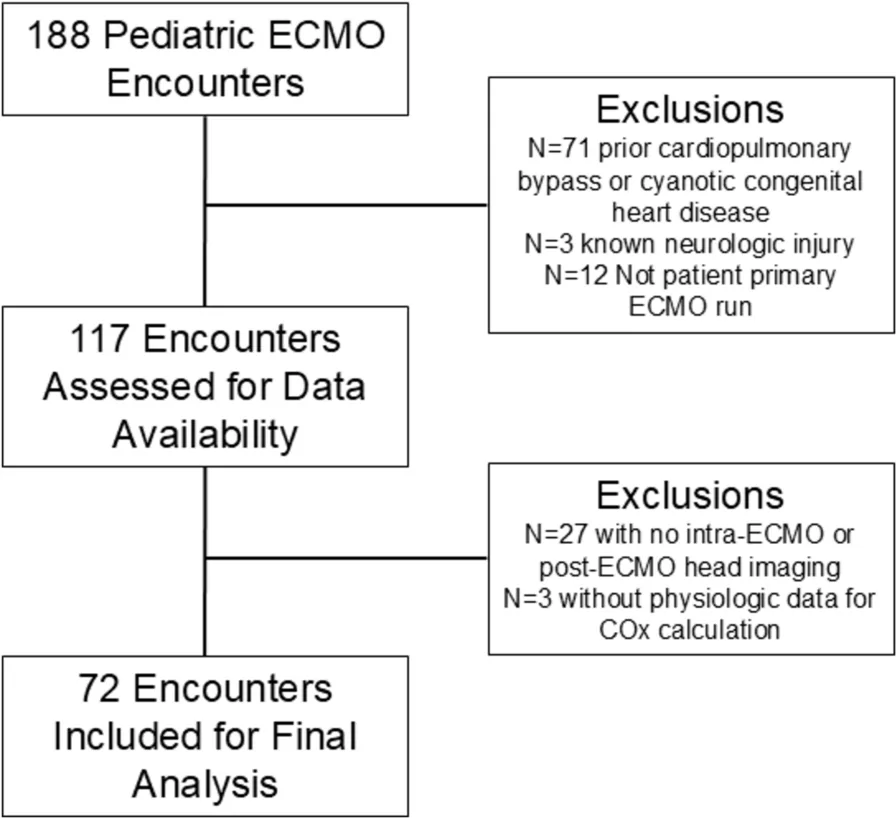
Patient encounters with total number of encounters screened and reasons for exclusion resulting in studied population

Initially, the MAP at which the lower limit and upper limit of autoregulation occurred on the basis of plotting COx against MAP in 5 mmHg bins was employed, but we were unable to fit a U-shaped curve to these plots for most patients. In prior studies, measurements of COx based blood pressure limits of autoregulation occur during cardiopulmonary bypass or ECMO support. Our measurements spanned pre-ECMO and on-ECMO periods wherein dynamic changes in pulse pressure, blood flow, blood pressure, and cerebral autoregulation occurred, which may have altered the blood pressure thresholds at which impaired CAR occurred, preventing application of the standard U-shaped curve. Therefore, to quantify the relationship between hypo- or hypertension during impaired CAR and neurologic injury, we calculated the area of average MAP above or below the age-based 50th percentile MAP [24] in temporal windows where COx > 0.35. The area under the curve (AUC) was calculated as AUC = ∑^N^_*i* = 0_ (MAPi − MedianForAge) × ∆Time, where the index *i* runs over all temporal windows where COx > 0.35 and ∆Time = 5 min. Negative values, then, represent hypotension and positive values hypertension. The AUC is representative of the magnitude of blood pressure variation from the normal median during impaired CAR (Fig. 1). Similarly, the AUC for mean blood pressure regardless of COx was calculated. This was termed MAP AUC and was used to determine whether blood pressure alone was associated with neurologic injury regardless of impaired CAR measured by COx. Windows with missing data were excluded in both COx and AUC calculation.

### Statistical Analysis

The percentage of time with impaired CAR was calculated for the entire ECMO run. The ECMO run was then stratified into pre-ECMO (up to 24 h prior to ECMO cannulation), peri-cannulation (0–12 h after cannulation), and intra-ECMO time periods (12 h–14 days after ECMO cannulation). The percent time with impaired CAR for each child and median COx value in each period were calculated. Wilcoxon rank-sum (continuous variables) and Pearson chi squared (categorical variables) determined differences in time with impaired cerebral autoregulation between ECMO time periods and between children with and without significant neurologic injury, any neurologic injury, and mortality. A low number of children suffered severe neurologic injury (score 9 or greater); therefore, subsequent adjusted analysis used NIS as a continuous variable to better analyze differences in neurologic injury associated with the AUC metric.

The AUC was calculated for each patient. Linear regression was used to determine the association between AUC during the distinct ECMO epochs and neurologic injury. A multivariable linear regression model was fitted to identify whether AUC is associated with NIS after adjustment for clinical variables expected to impact CAR. To test the hypothesis that the relationship between AUC and neurologic injury varies by ECMO time periods, the model included an interaction term between AUC and study epoch (pre-ECMO, peri-cannulation, and intra-ECMO). The model was adjusted for age (months), type of ECMO (VA vs. VV), and primary diagnosis (respiratory, cardiac, or other).

As subgroup analyses, the population was stratified by type of ECMO (venovenous versus venoarterial) and age (infant < 12 months versus children) to assess differences in impaired CAR and neurologic injury in each subgroup. The association between time with impaired autoregulation and neurologic injury for distinct ECMO time periods was determined with linear regression. To assess the effects of blood pressure alone, the MAP AUC metric calculated over the entire ECMO encounter regardless of COx was stratified by ECMO time periods, and linear regression was used to test for association with NIS.

## Results

Among 188 patients who received ECMO during the study time frame, 72 encounters were included for analysis. Exclusions included 12 due to non-primary ECMO cannulation, 71 due to post-cardiopulmonary bypass or congenital heart disease, and 29 due to either lack of neuroimaging or monitoring with cerebral oximetry.

The baseline demographic and ECMO characteristics of the study population were calculated, with respiratory failure constituting the most common indication for ECMO (Table 1). The study population included 36 (50%) infants less than 12 months old. Anticoagulation was achieved with heparin in 70 (97.2%) of patients versus bivalirudin in 2 (2.8%). Elevated lactic acid was common, consistent with the expected level of illness in a pediatric ECMO population. Head CT was evaluated in 27 (37.5%) and brain MRI in 55 (76.5%). In this patient cohort, only nine (12.5%) children suffered SNI on head imaging. On head imaging, 30 (41.7%) children had non-severe injury. Therefore, a total of 39 (54.2%) children in the patient population had radiographic abnormalities indicating at least minor neurologic injury associated with ECMO.

**Table 1:** Baseline demographic and ECMO characteristics of the study population

Characteristic	Total, n = 72	SNI, n = 9	No SNI, n = 63
Male Sex	35 (48.6)	2 (22.2)	33 (52.3)
Age (months)	11.7 (0.1–135.1)*	54.1 (6.0–154.8)*	9.2 (0.1–133.3)*
Race			
White	23 (31.9)	3 (33.3)	20 (31.8)
African American/Black	17 (23.6)	3 (33.3)	14 (22.2)
Asian	4 (5.6)	0 (0)	4 (6.3)
Other	28 (38.9)	3 (33.3)	25 (39.7)
Ethnicity			
Hispanic	23 (34.4)	3 (33.3)	20 (31.8)
Non-Hispanic	39 (55.5)	6 (66.7)	33 (52.4)
Unknown	10 (13.9)	0 (0)	10 (15.9)
ECMO Type			
Veno-Venous	25 (34.7)	0 (0)	25 (39.7)
Veno-Arterial	47 (65.3)	9 (100)	38 (60.3)
Time on ECMO (hours)	126.1 (93.2–242.2)*	103.5 (96.0–139.8)*	140.8 (92.8–263.8)*
ECMO Indication			
Cardiac	21 (29.2)	4 (44.4)	17 (27.0)
Respiratory	41 (56.9)	2 (22.2)	39 (61.9)
Other	10 (13.9)	3 (33.3)	7 (11.1)
Anticoagulation			
Heparin	70 (97.2)	9 (100)	61 (96.8)
Bivalirudin	2 (2.8)	0	2 (3.2)
Highest Lactate	4.3 (2.1–8.3)*	5.5 (2.8–10.2)*	3.7 (1.8–7.5)*
Death Prior to PICU Discharge	10 (14.1)	3 (33.3)	7 (11.3)

PICU, pediatric intensive care unit

^*^Median value with interquartile ranges

The total time of recorded data for the patient cohort was 15,067.6 h. Time-matched cerebral oximetry and blood pressure data were available for 11,383.4 h; therefore, the COx index could be calculated for the patient cohort for 75.5% of time. The median monitoring time for each patient was 138.3 h. Importantly, pre-ECMO data were only available for 25 (34.7%) encounters wherein a median of 6.9 h [interquartile range (IQR) 3.0–11.3] were monitored. Peri-cannulation data were available for 58 (81%) encounters with median monitoring time of 8.2 h (IQR 6.2–9.7). Intra-ECMO data were available in all encounters for a median of 110.3 h (IQR 80.1–128.4).

**Fig. 2:**
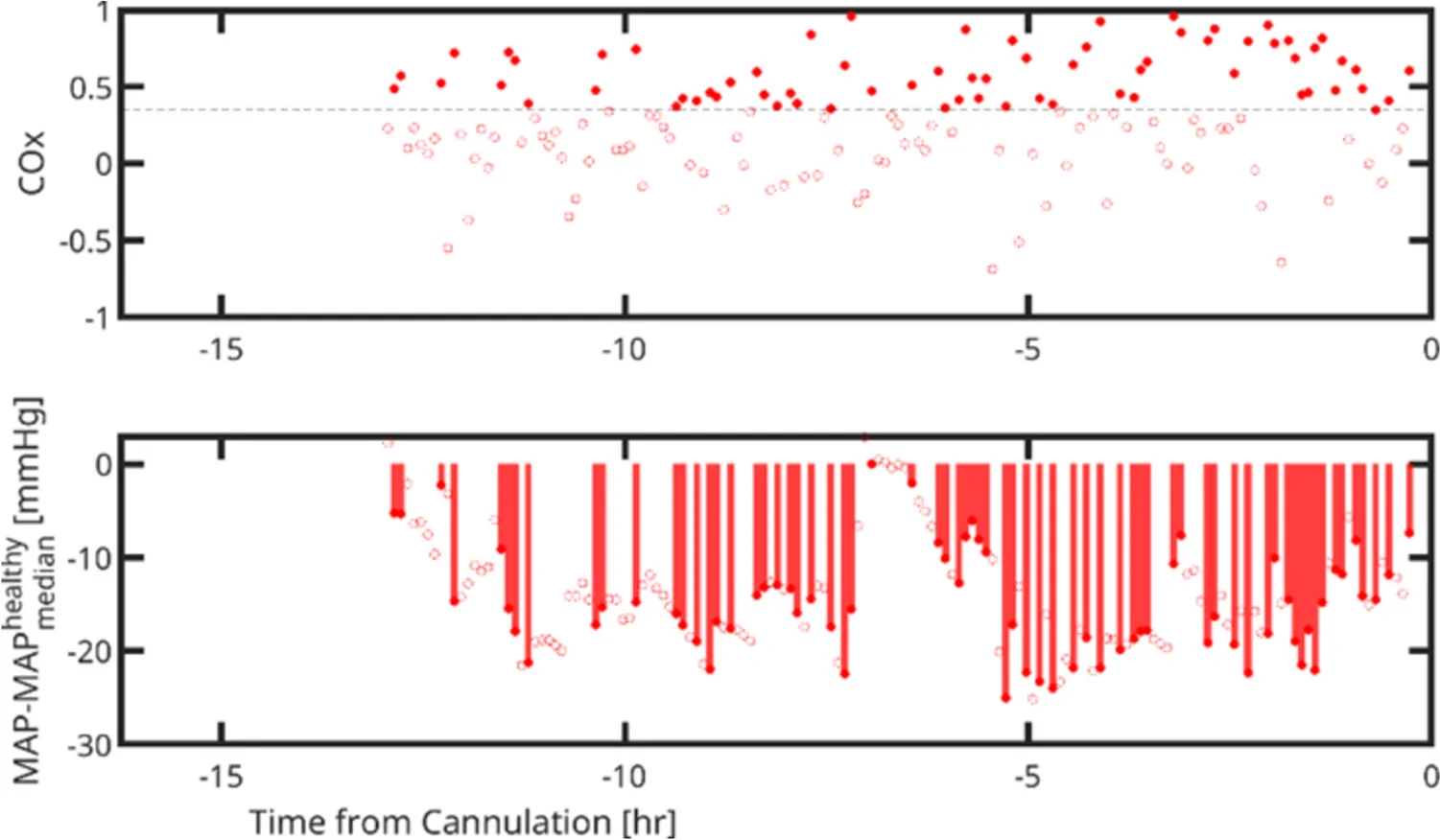
Example pre-ECMO time course (time 0 is ECMO cannulation) in subject with a significant burden of impaired CAR. COx is depicted in the upper window, with the dotted line representing the 0.35 cutoff. The bottom window depicts the difference between MAP and 50th centile standard blood pressure in solid red lines during time periods with COx > 0.35; the summation of these blood pressure differences constitutes AUC in the pre-ECMO time period for this patient

A histogram demonstrated a skewed distribution of total time with impaired cerebral autoregulation. Most patients were dysregulated 10–20% of the time (Fig. 2). When stratified by phase of ECMO, time with impaired CAR was significantly higher in the pre-ECMO time period than in the peri-cannulation time (28.2% versus 17.1%, *P* < 0.01). When restricted to only the 25 children with both pre- and peri-cannulation COx calculations, the difference in time with impaired cerebral autoregulation was still significant (28.2% vs. 18.3%, *P* = 0.008).The median COx value was 0.12 (IQR 0.06, 0.18) in the pre-ECMO time period versus 0.03 (IQR − 0.04, 0.08) and 0.04 (IQR − 0.02, 0.10) in the peri-cannulation and intra-ECMO time periods, respectively.

**Fig. 3:**
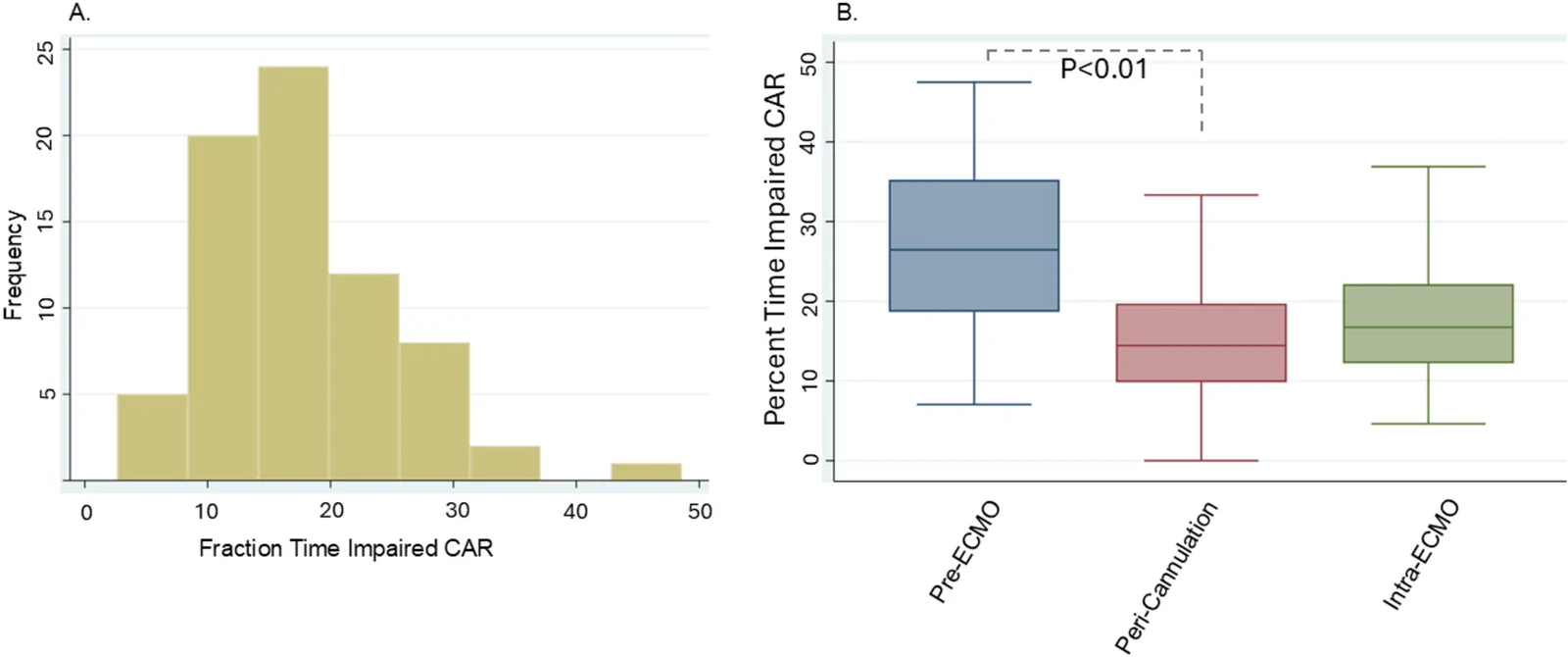
(A) Histogram depicting the distribution of percent time with impaired CAR for the entire study population. (B) Box plot depicting percent time with impaired CAR stratified by ECMO time periods

The percent time with impaired CAR throughout ECMO care was not associated with SNI, any neurologic injury, or mortality (Table 2). Given the significant differences between pre-ECMO impaired cerebral autoregulation and other phases of ECMO care, neurologic injury was assessed by phase of ECMO. There was no difference in median NIS between children with available monitoring data pre-ECMO (3, IQR 1 − 7) and those without (3, IQR 0–8), *P* = 0.95. The pre-ECMO burden of impaired cerebral autoregulation was higher in children with SNI and any neurologic injury (Fig. 3), though it did not reach the threshold for statistical significance (*P* = 0.48 and *P* = 0.19, respectively).

**Table 2:** Median percent time with impaired CAR with interquartile ranges stratified by SNI

	Percent Time Impaired CAR	P Value
Severe Neurologic Injury		0.38
Yes (n = 9)	16.6 (13.1–21.6)	
No (n = 63)	12.2 (10.9–24.6)	
Any Neurologic Injury		0.09
Yes (n = 39)	17.0 (15.4–23.7)	
No (n = 33)	15.1 (11.4–19.7)	
Mortality		0.19
Yes (n = 10)	16.8 (8.3–18.5)	
No (n = 62)	15.2 (13.1–21.8)	
ECMO Time Period		< 0.01
Pre-ECMO (n = 25)	26.5 (18.7–35.2)	
Peri-Cannulation (n = 58)	14.8 (9.8–20.0)	
Intra-ECMO (n = 72)	16.6 (12.2–22.1)	

The significance of differences by outcome were determined with the Wilcoxon rank-sum test. CAR, cerebral autoregulation; SNI, severe neurologic injury

Analysis of the magnitude of blood pressure abnormalities occurring during impaired CAR using AUC demonstrated that most children experienced hypotension prior to ECMO cannulation and, thus, had negative AUC values. SNI was significantly associated with pre-ECMO AUC (*P* = 0.006; Fig. 3). Peri-cannulation and intra-ECMO AUC were not associated with SNI, any neurologic injury, or mortality.

The multivariable linear regression model determined a significant association between lower AUC in the pre-ECMO time period (indicative of hypotension during impaired CAR) and increased NIS (coefficient =  − 0.008; 95% CI − 0.015, − 0.001; *P* = 0.03; *R*^2^ = 0.194). The association was attenuated after ECMO cannulation. VA ECMO mode was independently associated with higher NIS (coefficient = 3.44; 95% CI 0.97, 5.91; *P* = 0.007). Age and ECMO indication were not associated with NIS (Table 3).

**Table 3:** Multivariable linear regression analysis of AUC association with NIS

Variable	Coefficient	(95% CI)	P
Pre-ECMO AUC	-0.008	-0.015, -0.001	.03
Peri-ECMO × AUC	0.007	-0.001, 0.016)	.10
Intra-ECMO × AUC	0.008	0.001, 0.015	0.02
Age (months)	-0.007	-0.020, 0.006	.31
VA ECMO Mode (vs VV)	3.44	0.97, 5.91	0.007
Diagnosis (Ref: Respiratory)			
Cardiac	1.16	-1.43, 3.74	.38
Other	2.78	-0.49, 6.05	.10

AUC for the difference between a patient’s mean arterial blood pressure and age-based normal mean arterial blood pressure when COx > 0.35. The negative coefficient indicates an association of higher NIS with lower AUC in the pre-ECMO period. Positive AUC × epoch interaction terms indicate attenuation of the AUC–NIS relationship after cannulation. AUC, area under the curve; CI, confidence interval; NIS, neurologic injury score; VA, venoarterial; VV, venovenous

**Fig. 4:**
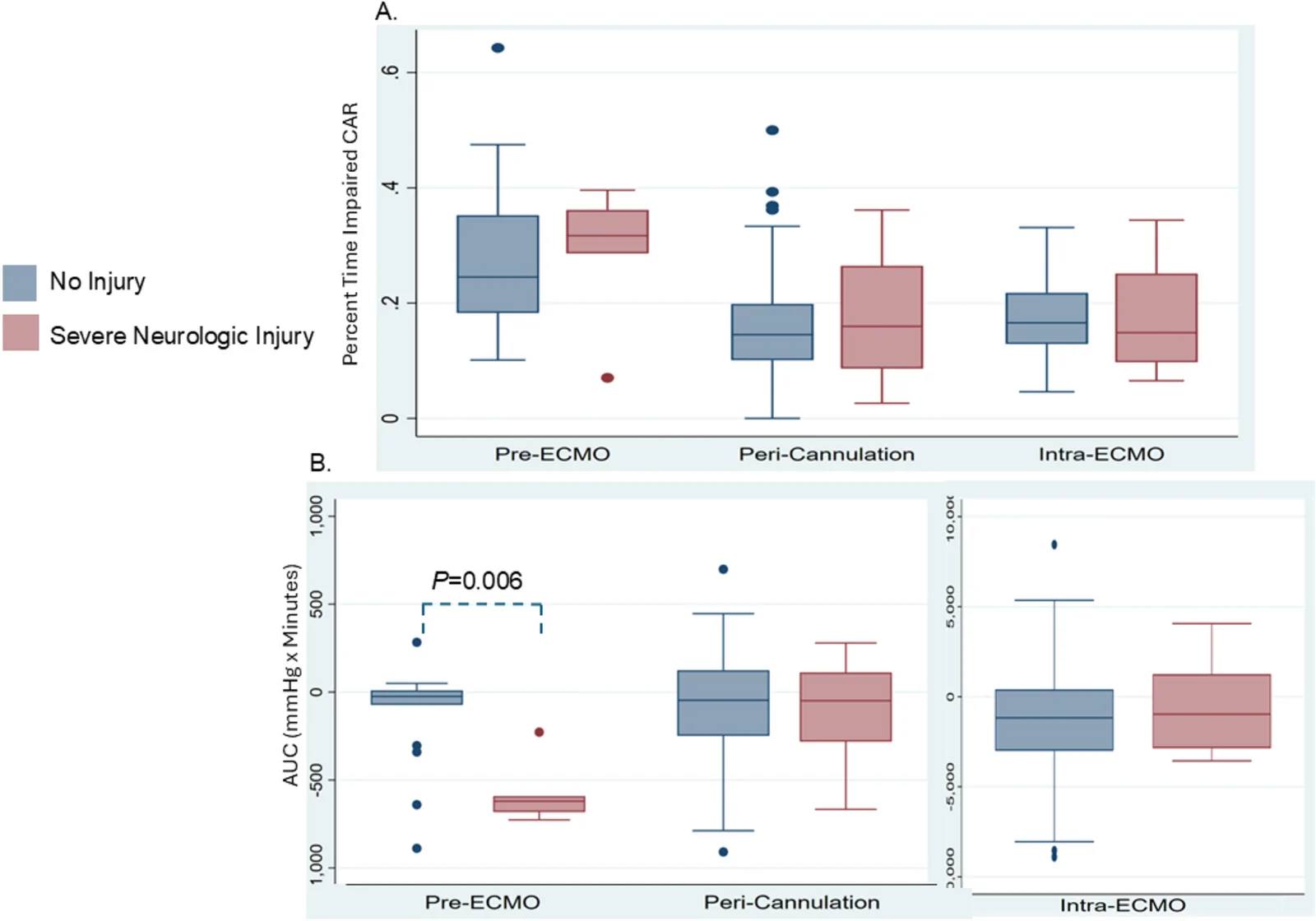
Box plots depict the median values and interquartile ranges of fractional time of impaired CAR (A) and AUC (B). Data were stratified by occurrence of severe neurologic injury (A). The AUC was significantly lower among children with severe neurologic injury, indicating impaired CAR in combination with hypotension was associated with neurologic injury

**Fig. 5:**
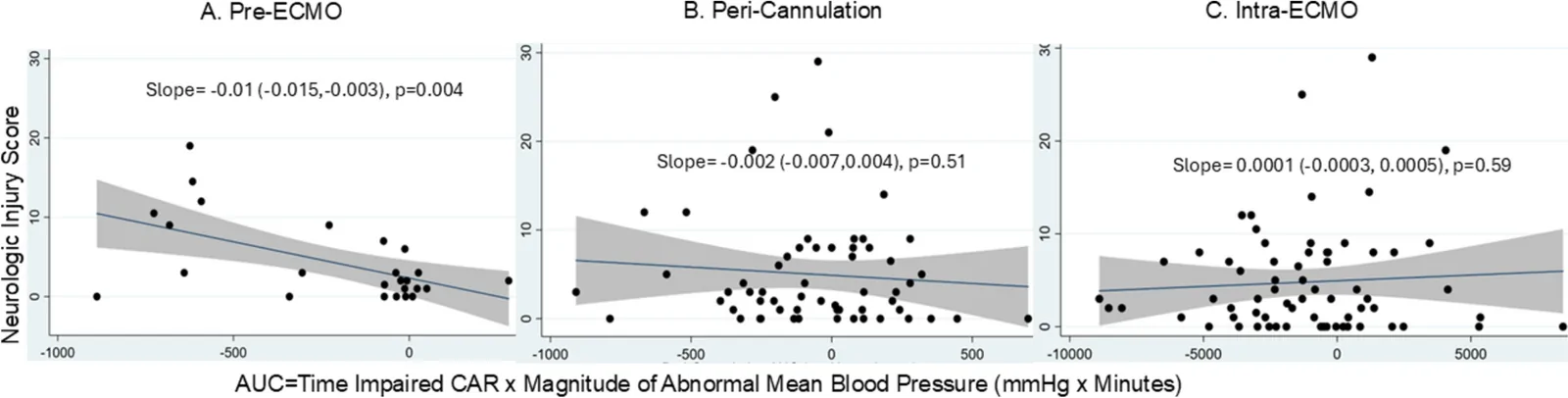
Linear regression plots with each dot representing the AUC of a distinct patient during (A) pre-ECMO, (B) peri-cannulation, or (C) intra-ECMO phases of care. Lower AUC, indicating impaired CAR in combination with hypotension, during the pre-ECMO time period is associated with increased neurologic injury

Univariate linear regression of pre-ECMO AUC demonstrates a significant association with neurologic injury (coefficient − 0.01; − 0.02, − 0.003; *P* = 0.004, *R*^2^ = 0.31) wherein higher magnitudes of hypotension during impaired autoregulation state were associated with higher neurologic injury scores (Fig. 4). Alternatively, neurologic injury scores were not associated with peri-cannulation or intra-ECMO AUC (Fig. 4). Linear regression of the mean blood pressure AUC, which was calculated to analyze blood pressure alone regardless of COx, demonstrated no significant association with neurologic injury in the pre-ECMO (*P* = 0.18), peri-cannulation (*P* = 0.60), or intra-ECMO (*P* = 0.54) time periods.(Figure 5)

After stratifying the study population by receipt of VA or VV ECMO, increased median NIS was noted for VA ECMO (6, IQR 1–9) as compared with VV ECMO (1, IQR 0–3, *P* < 0.001). No differences in percent time with impaired CAR was detected in any ECMO time period between VA and VV ECMO encounters (Supplementary Table S1A). However, AUC during the pre-ECMO time period was significantly associated with NIS for VA ECMO (coefficient =  − 0.01; − 0.02, − 0.002; *P* = 0.02, *R*^2^ = 0.34), but not for VV ECMO (coefficient =  − 0.001; − 0.005, 0.003; *P* = 0.55, *R*^2^ = 0.05) (Supplementary Table S1B).

Similarly, the infant population was assessed separately, and no difference in burden of impaired CAR was detected between infants < 12 month and children (Supplementary Table S2A). There was no significant difference between NIS for infants versus children. However, no infants died prior to hospital discharge, and severe neurologic injury occurred in 3 (8.3%) infants versus 6 (16.7%) children. Linear regression determined that the AUC during the pre-ECMO time period was associated with NIS in both infants and children but was not associated with NIS in any other time period (Supplementary Table S2B).

## Discussion

We used physiological data to estimate time with impaired CAR during separate phases of pediatric ECMO care. We report a significantly higher burden of impaired CAR that occurred before ECMO cannulation. After cannulation, impaired CAR was similar during the 12-h post-cannulation and the rest of ECMO care. A prior study that assessed cerebral oximetry index only after ECMO cannulation found an association between increased time with impaired CAR and neurologic complications [18]. We failed to find an association between intra-ECMO impaired cerebral autoregulation and neurologic injury assessed by standardized scoring of head imaging.

However, pre-ECMO impaired CAR in combination with hypotension was significantly associated with neurologic injury evaluated as both a continuous variable and as a dichotomized variable at a threshold previously shown to be associated with impaired functional neurologic outcomes. In isolation, blood pressure differences from age-based standards were not associated with neurologic injury in any ECMO time period. Our results indicate that the simultaneous occurrence of impaired CAR and abnormal blood pressure (particularly hypotension) before ECMO increases the risk for neurologic injury, possibly beyond the risk of abnormal blood pressure alone. The relationship between pre-ECMO AUC and neurologic injury was primarily detected among patients cannulated to VA ECMO. The reasons for impaired CAR and associated risk may differ between patients with VA ECMO and those with VV ECMO, requiring further studies to define relationships. No differences in impaired CAR, AUC, or radiographic neurologic injury were detected between infants and children. Infants suffered less severe neurologic injury, and no infants died prior to pediatric ICU (PICU) discharge. Our results may lack the power to detect differences in impaired CAR and related radiographic neurologic injuries in infants versus children. However, the significant association between the pre-ECMO AUC metric occurred in both infants and older children. The pre-ECMO association of impaired CAR and AUC with injury may be influenced by selection bias due to clinician selection of higher risk patients for NIRs monitoring. However, no difference in neurologic injury score was detected between those with and without pre-ECMO monitoring. Nonetheless, these findings should be interpreted as hypothesis-generating rather than defining causation.

There are several implications for these findings. First, they may indicate that the risk of neurologic injury occurs before ECMO cannulation. The specific clinical criteria currently used to deploy ECMO vary on the basis of clinical and center-specific factors. The burden of impaired cerebral autoregulation may serve as another, more specific, marker of cerebral health to help guide clinicians. Further, ECMO initiation led to rapid, relatively large changes in CAR. These rapid changes may impact cerebral blood flow during the cannulation time period and serve as a modifiable risk factor in ECMO-related neurologic injury. Importantly, we are unable to establish the timing of neurologic injuries in this study. While our data may indicate that in this cohort neurologic injuries may occur before or during ECMO cannulation, that does not necessarily mean that impaired CAR during ECMO is not associated with intra-ECMO injuries. Our cohort may not include sufficient numbers of patients who suffered neurologic injury during ECMO to sufficiently characterize intra-ECMO impaired CAR in relation to injuries. Additionally, impaired CAR may occur secondary to injury rather than serving as an indicator for risk of subsequent injury. The temporal relationship between impaired CAR and neurologic injury requires further investigation.

These results support the growing body of evidence that rapid peri-cannulation changes in physiologic parameters may result in poor neurologic outcomes. Larger changes in blood carbon dioxide levels after ECMO cannulation have been associated with neurologic complications in neonatal, pediatric, and adult patients with ECMO [15, 17, 28]. The putative mechanism of injury development is fluctuation in cerebral blood flow caused by changes in carbon dioxide. Importantly, the effect of carbon dioxide levels on cerebral blood flow may depend on complex interactions with systemic blood pressure and patient or disease-specific factors. A study evaluating COx and partial pressure of carbon dioxide (pCO2) found relative improvement in COx with mild hypercapnia, except in the case of hypotension, when elevated pCO2 seemed to negatively influence COx determined cerebral autoregulation [29]. Our results suggest that hypotension occurring during impaired autoregulatory state may be particularly harmful. Currently, clinical management of factors including carbon dioxide removal, blood pressure, and ECMO pump flows lack feedback from real-time monitors of cerebral autoregulation. COx may serve as an intervenable monitor of cerebral autoregulation. However, further studies are needed to verify the association of COx with clinical outcomes and elucidate the relationship between modifiable clinical factors and COx.

Our study was limited in several ways. We were restricted to analysis of retrospective data on relatively few patients in whom severe neurological injury occurred. Additionally, pre-ECMO data were available in 25 (34.7%) encounters, limiting statistical power. Additionally, the patients who had pre-ECMO data available for COx calculation may have had different clinical characteristics than those cannulated prior to establishing cerebral oximetry or arterial line monitoring, leading to selection bias. Missing NIRS or arterial line monitoring data may have been more likely during the critical pre-ECMO and peri-cannulation epochs leading to unmeasured impaired CAR, which may have prevented detection of important associations between impaired CAR and clinical outcomes. The available physiologic data were available only at the 0.2 Hz sampling rate, which is below the 20 Hz sampling rate that has been recommended for optimal raw data extraction. [30]. The available data of 0.2 Hz may, then, have prevented preservation of phase and amplitude fidelity of slow vasogenic oscillations. Our patient selection criteria restricted analysis to children in whom head imaging occurred, which may have created selection bias toward children who had clinical exam concerns leading to intra-ECMO CT or, conversely, may have biased toward a population who survived to decannulation and underwent brain MRI.

## Conclusions

This is the first report we are aware of to analyze the pre-ECMO cannulation burden of impaired CAR measured by COx. The large difference in impaired CAR between the pre-ECMO and peri-cannulation periods indicates variable cerebral blood flow states. We did not find an association between overall time with impaired cerebral autoregulation and neurological injury on head imaging. However, pre-ECMO impaired CAR combined with the magnitude of abnormal blood pressure was associated with neurologic injury. Our results support further research to determine what impact impaired CAR has on neurologic outcomes in pediatric patients with ECMO and whether modifiable clinical factors may ameliorate impaired CAR.

## Supplementary Information

Below is the link to the electronic supplementary material.

Supplementary file1 (DOCX 18 KB)

Supplementary file2 (DOCX 17 KB)
