# Supplementary material for: Impaired Cerebral Autoregulation in Pediatric Patients with ECMO: A Retrospective Analysis of ECMO Time Periods and Association with Neurologic Injury
Source: Neurocrit Care. 2026 May 18;45(2):750–9. doi: 10.1007/s12028-026-02531-1 (PMC13642396; doi:10.1007/s12028-026-02531-1)
Supplement: Supplementary file 1 [file 12028_2026_2531_MOESM1_ESM.docx]

|  | Infant <12mo, n=36 | Child >12mo, n=36 | P |
| --- | --- | --- | --- |
| total monitored time (hours) | 131.2 (102.9, 169.1) | 136.5 (91.4, 267.3) | 0.34 |
| Total Percent time impaired car | 16.5 (12.8, 20.7) | 17.5 (11.7, 22.8) | 0.69 |
| Pre-ECMO percent time impaired CAR | 26.5 (21.3, 39.6) | 26.1 (18.3, 34.3) | 0.44 |
| Peri-Cannulation percent time impaired CAR | 13.1 (10.4, 19.1) | 16.2 (9.8, 26.5) | 0.43 |
| Intra-ECMO percent time impaired CAR | 17.9 (11.7, 23.2) | 16.2 (12.4, 20.2) | 0.44 |
| VA ECMO | 25 (69.4) | 22 (61.1) | 0.46 |
| Death Prior to PICU Discharge | 0 (0.0) | 10 (27.8%) | 0.001 |
| NeuroInjury Score | 3.00 (1.0, 7.0) | 2.5 (0, 8.0) | 0.69 |

**Supplemental Table 2A**: Univariate Associations Stratified by Infant versus Child

**Supplemental Table 1B**: Linear Regression Determined Association of Neurologic Injury Score (NIS) with AUC Stratified by Infant versus Child

|  | Infant <12mo (coefficient, CI) | P | Child >12mo (coefficient, CI) | P |
| --- | --- | --- | --- | --- |
| Pre-ECMO | -0.03 (-0.05, -0.006) | 0.02 | -0.01 (-0.02, -0.002) | 0.02 |
| Peri-Cannulation | 0.0006 (-0.01, 0.01) | 0.91 | -0.003 (-0.01, 0.004) | 0.39 |
| Intra-ECMO | 0.001 (-0.0003, 0.002) | 0.15 | -0.0001 (-0.0005, 0.001) | 0.97 |
