# Supplementary material for: Impaired Cerebral Autoregulation in Pediatric Patients with ECMO: A Retrospective Analysis of ECMO Time Periods and Association with Neurologic Injury
Source: Neurocrit Care. 2026 May 18;45(2):750–9. doi: 10.1007/s12028-026-02531-1 (PMC13642396; doi:10.1007/s12028-026-02531-1)
Supplement: Supplementary file 2 [file 12028_2026_2531_MOESM2_ESM.docx]

|  | VV ECMO, n=25 | VA ECMO, n=47 | P |
| --- | --- | --- | --- |
| Total Percent time impaired car | 16.8 (14.6, 24.7) | 15.9 (11.0, 20.8) | 0.07 |
| Pre-ECMO percent time impaired CAR | 15.1 (11.1, 20.4) | 14.7 (9.0,18.5) | 0.77 |
| Peri-Cannulation percent time impaired CAR | 17.1 (14.4, 20.3) | 16.2 (11.8, 20.3) | 0.81 |
| intra-ECMO percent time impaired CAR | 16.7 (13.8, 24.2) | 12.1 (12.2-21.2) | 0.09 |
| Age (Years) | 1.80 (0.20,11.30) | 0.30 (0.00,1.20) | 0.25 |
| Death Prior to PICU Discharge | 3 (12.0%) | 7 (14.9%) | 0.71 |
| NeuroInjury Score | 1.00 (0, 3.0) | 6.00 (1.0, 9.0) | <0.001 |

**Supplemental Table 1A**: Univariate Associations Stratified by Type of ECMO

**Supplemental Table 1B**: Linear Regression Determined Association of Neurologic Injury Score (NIS) with AUC Stratified by Type of ECMO

|  | VV ECMO (coefficient, 95% CI) | P | VA ECMO (coefficient, 95% CI) | P |
| --- | --- | --- | --- | --- |
| Pre-ECMO | -0.001 (-0.005, 0.003) | 0.55 | -0.01 (-0.02, -0.002) | 0.02 |
| Peri-Cannulation | -0.002 (-0.004, 0.001) | 0.22 | -0.0001 (-0.01, 0.01) | 0.98 |
| Intra-ECMO | -0.0002 (-0.0004, 0.0003) | 0.10 | 0.0004 (-0.0003, 0.001) | 0.09 |
